# Ethnic-specific reference range affects the efficacy of quadruple test as a universal screening for Down syndrome in a developing country

**DOI:** 10.1371/journal.pone.0251381

**Published:** 2021-05-13

**Authors:** Savitree Pranpanus, Ounjai Kor-anantakul, Thitima Suntharasaj, Chitkasaem Suwanrath, Tharangrut Hanprasertpong, Ninlapa Pruksanusak, Chusana Petpichetchian, Manaphat Suksai, Natthicha Chainarong, Rapphon Sawaddisan

**Affiliations:** Department of Obstetrics and Gynecology, Faculty of Medicine, Prince of Songkla University Hat Yai, Hat Yai, Songkhla, Thailand; University of Mississippi Medical Center, UNITED STATES

## Abstract

**Objective:**

To evaluate the efficacy of the quadruple test for potential use as a Thai national policy for Down syndrome (DS) screening and establish an accurate equation for risk estimation of Down syndrome based on gestational age, weight and the ethnic-specific reference range of our population.

**Methods:**

A prospective study was conducted on singleton pregnancies at 14 to 21 weeks of gestation to evaluate the efficacy of quadruple DS screening using the automatically calculated Western European descent factor (WF) in our population and the impact of screening using a specific Thai ethnic factor as well as to establish an equation for the risk estimation of DS based on gestational age, weight and a local Thai ethnic factor to correct for the impact of ethnic factor on the screening efficacy.

**Results:**

Of a total of 5,515 women, 12 cases of DS and 8 cases of other aneuploidies were found. The detection rate, false positive rate and specificity were 75.0%, 9.1% and 90.9%, respectively, by automatic calculation with the widely used WF; the screening efficacy was lower when used in Asian populations than in other studies. The best-fitted regression equation of serum quadruple screening of AFP, free β-hCG, uE3 and inhibin A was established by adjustment for gestational age (GA) in days, maternal weight and our Thai-specific ethnic reference range which was created for this study. Calculations with our Thai-specific ethnic model gave a better detection rate of 83.3%, a false positive rate of 9.6% and specificity of 90.4%.

**Conclusion:**

The serum quadruple test had a lower detection rate than expected when the risk estimation was based on the WF reference range. The serum quadruple test using WF had significantly different levels when corrected with our ethnic-specific factor. Using our local ethnic specific model could increase the detection rate of DS screening in Thailand with a minimal increase in false positive rates. Our findings indicate that DS screening should be adjusted with an appropriate individual ethnic factor when used for national screening.

## Introduction

Despite advances in prenatal screening, Down syndrome is still a problematic disease, particularly in developing countries. Down syndrome (DS) is the most common form of trisomy with an overall incidence of 1.4:1,000 live births [1]. Children with DS need special care because of mental retardation and the co-morbidities associated with congenital malformation, which has a huge impact on developing countries all over the world [2, 3]. Although there is a more accurate cell-free fetal DNA test, due to the high cost of this test, the quadruple test is still being used for fetal DS screening, especially in low-resource countries [4–6]. Many studies have confirmed the screening efficacy of this test in developing countries with an acceptable detection rate for DS screening [7–10]. Because this test is convenient and not costly, it is appropriate for pregnant women who present later than the first trimester and for health care services that lack expertise in ultrasound to measure nuchal translucency or evaluate the second trimester sonomarkers of DS.

Although maternal serum quadruple screening detection rates of between 75 and 85% have been reported [10–15], these studies also found different false positive screening rates of 5 to 14%, which can lead to unnecessarily high numbers of invasive procedures and high laboratory costs for karyotyping, which are burdensome for low-resource countries. One of the reasons for the high false positive rates is the ethnic factor. Many studies have reported that using an incorrect ethnic factor with different reference ranges of serum markers leads to higher numbers of false positives. For example, using the Western European descent factor (WF), which is programmed into most automatic testing machines, to calculate DS risk in Thailand was found to have false positive rates as high as 13% in the triple test compared with an average false positive rate of 7.8% when using the ethnic-specific Thai factor (TF) in Thailand [7]. However, there are limited data regarding the efficacy and false positive rate in the Thai population for the quadruple test, which the National Thai Health Policy has selected as the universal DS screening test for pregnant Thai women.

In this study, our aim was to evaluate the quadruple test’s actual efficacy, which has been conducted free of charge for many years, as a Thai national policy for DS screening. We also aimed to investigate the effects of the ethnic-specific reference range, which we surmised may affect the quadruple screening’s efficacy and false positive rate, as there are still a lack of data regarding whether we should use the WF automatically calculated by the serum analysis machine or our ethnic factor for the quadruple test. If there is an impact on the detection or false positive rates from the automatic WF values, it would also affect the cost of newborn care for DS and cause a higher invasive procedures rate, which would add to the already extremely high budget for national health care across the country. This could also be why other developing countries support using their own locally developed reference ranges and ethnic factors for the quadruple test.

## Materials and methods

This study was conducted prospectively between January 2012 and 2019. Pregnant women were recruited from Songklanagarind Hospital and Hat Yai Hospital, the two main tertiary-care centers in southern Thailand. Ethical approval was given by the Faculty of Medicine’s Institutional Review Board, Prince of Songkla University. All who participated in this study were informed of the study design and aims and gave their written informed consent. From 2012 to 2014, werecruited pregnant women from Songklanagarind Hospital, and then after 2015 we recruited patients who had undergone the quadruple screening offered by the Thai Government National Down Syndrome Screening Policy, which was provided free of charge. However, the same laboratory setting was used for the test analyses in both periods so this had no impact on the data quality. All pregnant women who attended the antenatal clinics in the participating hospitals with gestational age between 14 and 21 weeks were enrolled. The inclusion criteria were 1) Thai ethnicity, and 2) singleton pregnancy. The exclusion criteria were 1) miscarriage during the study, 2) lost to follow-up, 3) fetal anomaly, and/or 4) chromosomal abnormality other than DS. All blood samples for the quadruple test were collected at 14 to 21 weeks of gestation and sent to the biochemistry lab at Songklanagarind Hospital. The turnaround time for the screening results was two weeks.

All patients’ basic pregnancy, clinical and demographic data were recorded, including age, body weight, body mass index (BMI), smoking status, pregnancy history, use of assisted reproductive technologies, and other medical conditions. Gestational ages were estimated by ultrasound as early as possible before 20 weeks of gestation to correct the gestational age. The maternal serum levels of α-fetoprotein (AFP), free β-human chorionic gonadotropin (free β-hCG), unconjugated estriol (uE3) and inhibin A were measured by a KRYPTOR compact PLUS (Thermo Fisher Scientific, Hennigsdorf, Germany) measuring device, and inhibin A was measured using TRACE (Time-Resolved Amplified Cryptate Emission; Ansh Labs, Webster, TX, USA) and Immunomat (Institut Virion\Serion, Würzburg, Germany) analyzers. The crude levels of all serum level markers were recorded, then all serum levels were automatically converted to multiples of the median (MoMs) with adjustment for maternal weight based on WF gestation-specific medians from the serum analyzer.

The risk adjustment for DS considered to be high was 1:250 or greater. Patients who had a high risk of a DS fetus based on a positive test were offered amniocentesis for prenatal diagnosis. However, some patients with a negative test result still chose to undergo an invasive diagnostic procedure due to maternal age-related risk. The chromosome results were retrieved for all patients who underwent amniocentesis. All patients included in this study delivered their babies in one of our institutions. To evaluate the neonatal karyotype, the neonatologist examined and recorded the condition of the babies. If the babies were suspected of Down syndrome without a prenatal diagnosis, a blood sample was taken for karyotyping.

## Statistical analysis

The data were analyzed using Stata v14.2. Continuous variables were calculated as median ± SD. The distribution of each maternal serum quadruple marker was examined by plotting against gestational age. Quantile regression was conducted using the natural logarithm values of AFP, β-hCG, uE3 and inhibin-A against centralized gestational age and centralized maternal weight together with quadratic terms if appropriate to obtain the best fitting model for each marker. Predicted median values were estimated from the regression equations and plotted as a surface against gestational age and body weight. Multiple-of-medians (MoMs) for each subject were then calculated from the gestational age and body weight-specific predicted medians. These Thai-specific median MoMs were applied to the machine-specific estimated risk (based on the WF model) to identify women considered as high DS risk (≥ 1 in 250). Finally, the distribution of the logarithms of MoMs based on local medians and those based on the WF medians were separately plotted against gestational age to distinguish between DS and normal fetuses.

## Results

**Fig 1:**
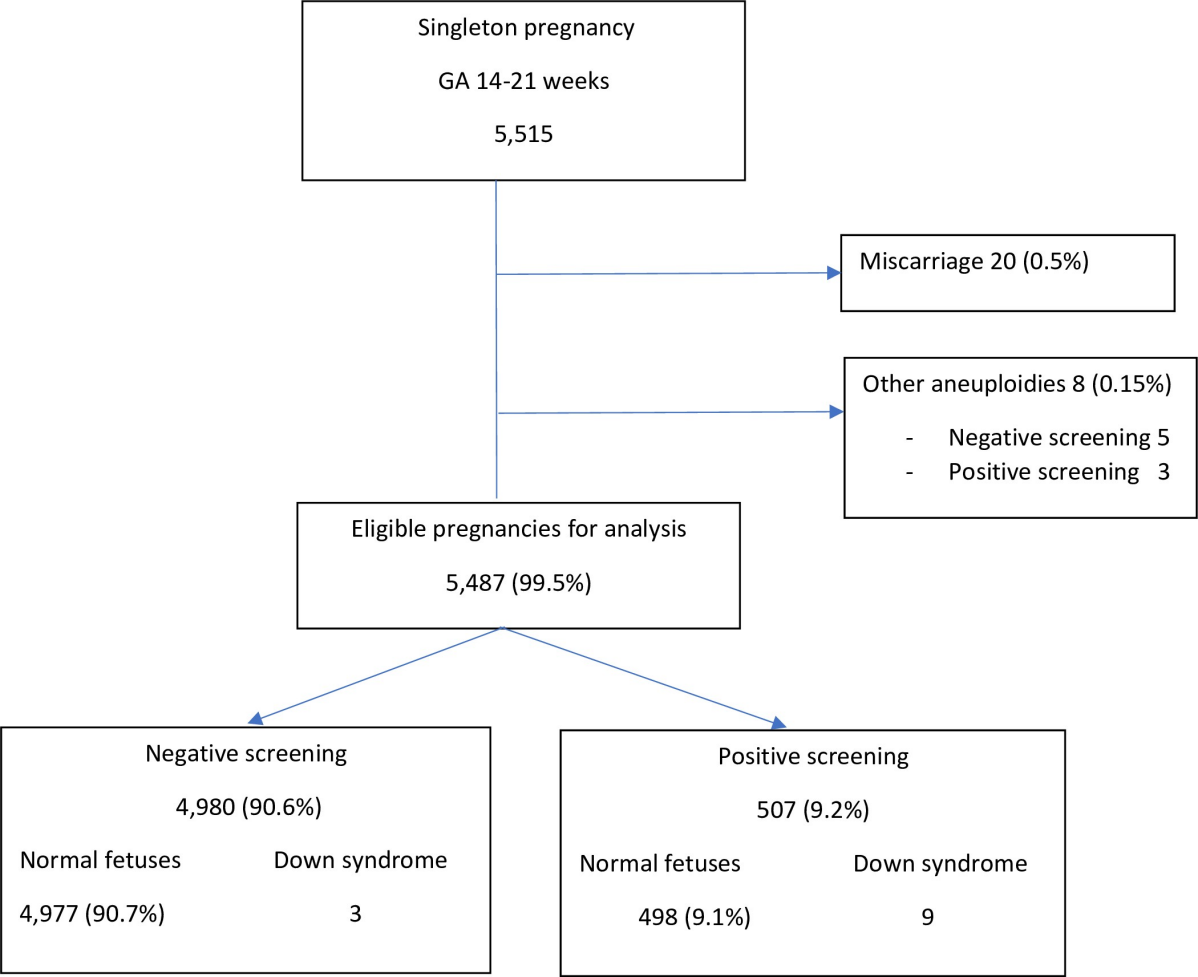
Flow chart of the pregnant women who underwent quadruple serum screening for Down syndrome.

During the study period, 5,515 pregnant women with a gestational age of 14 to 21 weeks who attended one of our antenatal clinics underwent quadruple screening (Fig 1). Twenty eight cases (0.5%) were excluded from the study, 20 for miscarriage and 8 for fetal chromosomal abnormalities other than DS (1 trisomy 13, 2 trisomy 18, 46,XY,t(1;14), 47,XX,+20[3]/46,XX[45], 45,X, 47,XXY and 47,XXX). The patients with trisomy 13 or 18 terminated their pregnancies, while the other aneuploidy cases carried on to term delivery. The remaining 5,487 (99.5%) cases were included in the analysis, with no cases lost to follow up after delivery. All of the participants delivered in one of the two study hospitals.

In our study, we found 9 cases of DS following screening, but at the end of the study there were 12 DS cases detected. Of all the participants included in this study, 507 (9.2%) had a positive screening result. Amniocentesis was performed in 474 participants: 376 (79.3%) were indicated by high-risk DS screening, and 98 (20.7%) were done based on maternal age-related risk and some sonomarkers indicative of possible DS were found. Three false negative results cases had DS risk less than 1:500. One of the three underwent amniocentesis owing to the presence of multiple sonomarkers for DS. The other two false negatives were revealed by karyotyping after delivery due to the newborns having physical examination characteristics suspicious for DS.

The demographic data of the participants are summarized in Table 1. The median gestational age on the day of screening was 16.4 weeks (115 days). The number of screenings was highest during the 15^th^ and 16^th^ weeks (38.7%). The median maternal age at screening was 29.1 years, and 11% of the participants were ≥ 35 years.

**Table 1:** Demographic data of the pregnant women who underwent quadruple maternal serum screening (5,515 women).

Parameter	Median (min, max)
1. Maternal age at screening, years	29.1 (14.0,46.0)
2. Maternal weight at sampling, kg	57.1 (31.5,165)
3. Gestational age at sampling, days	115 (98,145)
4. Gestation age at delivery, weeks	38 (15,42)
5. Birth weight, g	3,100 (210, 4878)
6. Parity, %	
• 0	55.0
• 1	36.6
• ≥ 2	8.4
7. Gestational age at screening, days	Number (%)
• 98–104	523 (9.5)
• 105–111	2,136 (38.7)
• 112–118	1,808 (32.8)
• 119–125	800 (14.5)
• 126–132	215 (3.9)
• 133–139	24 (0.4)
• 140–146	9 (0.2)

**Fig 2:**
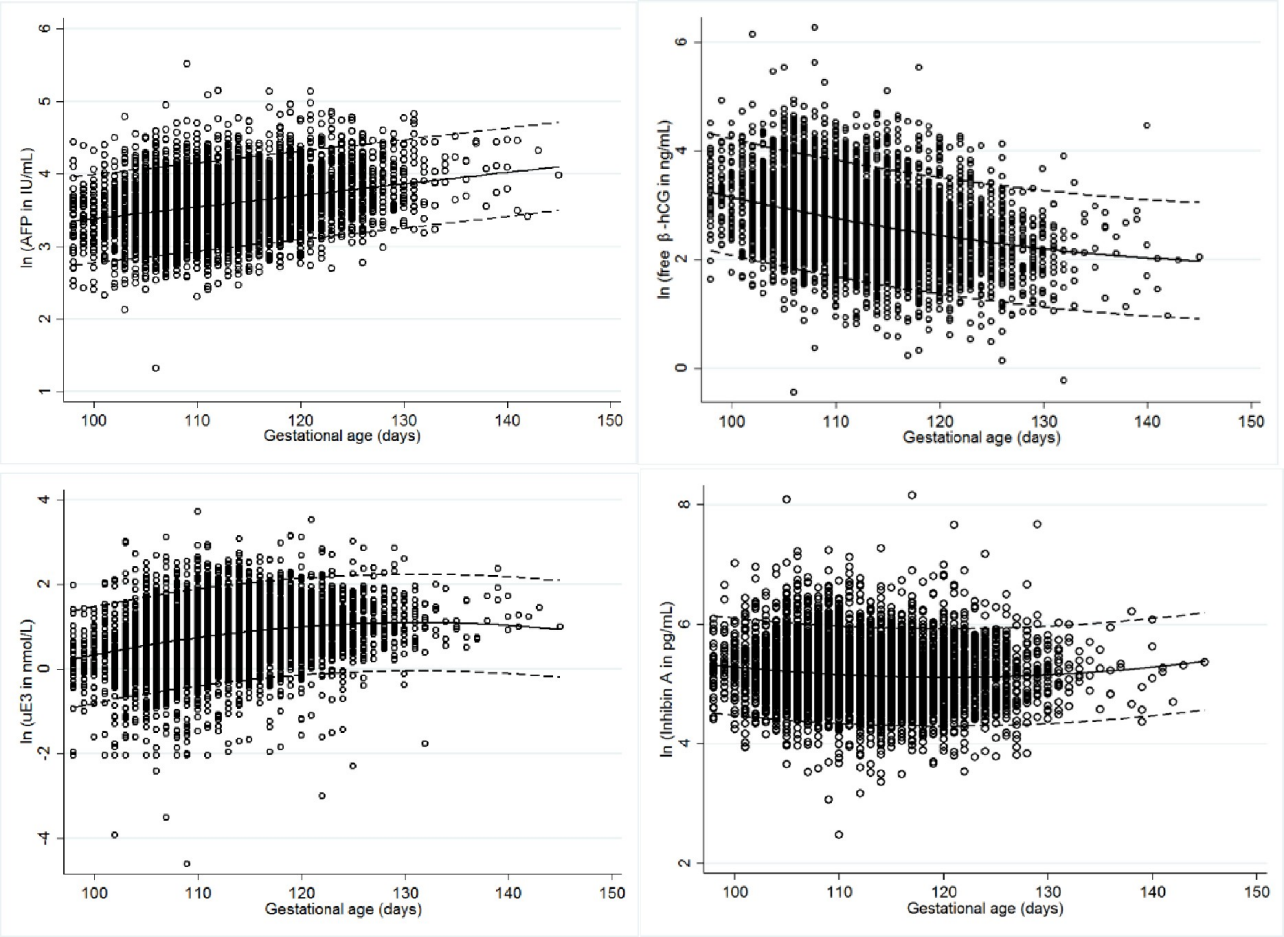
Scatter plots of the log regression of median levels of AFP, free β-hCG, unconjugated estriol (uE3) and inhibin A according to gestational age by days of normal fetuses. The straight lines show the tendency of the regression line across gestational age at the 5th, 50th and 95th percentiles.

The distribution of the logs of maternal serum AFP, free β-hCG, inhibin A and uE3 of normal fetuses according to the gestational age in days are shown in Fig 2. The scatter plots of the normal fetuses (Fig 2) show the normal distribution and trend of each serum throughout the gestational period of the screening. The AFP and uE3 levels tended to increase with advanced gestation, but uE3 plateaued after around 17 weeks. The free β-hCG level decreased throughout the screening period, while the inhibin A level decreased during the mid-period and then slightly increased during the late screening period.

Regression equations to predict the median concentration levels of the maternal serum quadruple markers according to gestational age (days) and maternal weight (kg) for calculation of our local specific MoMs during the screening period were constructed as follows:

$$Median\;AFP\;(IU/mL)=exp\;[0.0172(GAday-115)-0.0258(Wt-57)+0.000114{\left(GAday\right)}^{2}+3.2966]$$ (1)

$$Median\;free\;\beta -hCG\;(ng/mL)=exp\;[-0.120(GAday-115)-0.0249(Wt-57)+0.000105({Wt}^{2})+0.00381{\left(GAday\right)}^{2}]$$ (2)

$$Median\;uE3\;(nmol/L)=exp\;[0.195(GAday-115)-0.00495(Wt-57)-0.000743{\left(GAday\right)}^{2}+10.7]$$ (3)

$$Median\;inhibin\;A\;(pg/mL)=exp\;[-0.113(GAday-115)-0.00498(Wt-57)+0.000468{\left(GAday\right)}^{2}-1.0543]$$ (4)

**Fig 3:**
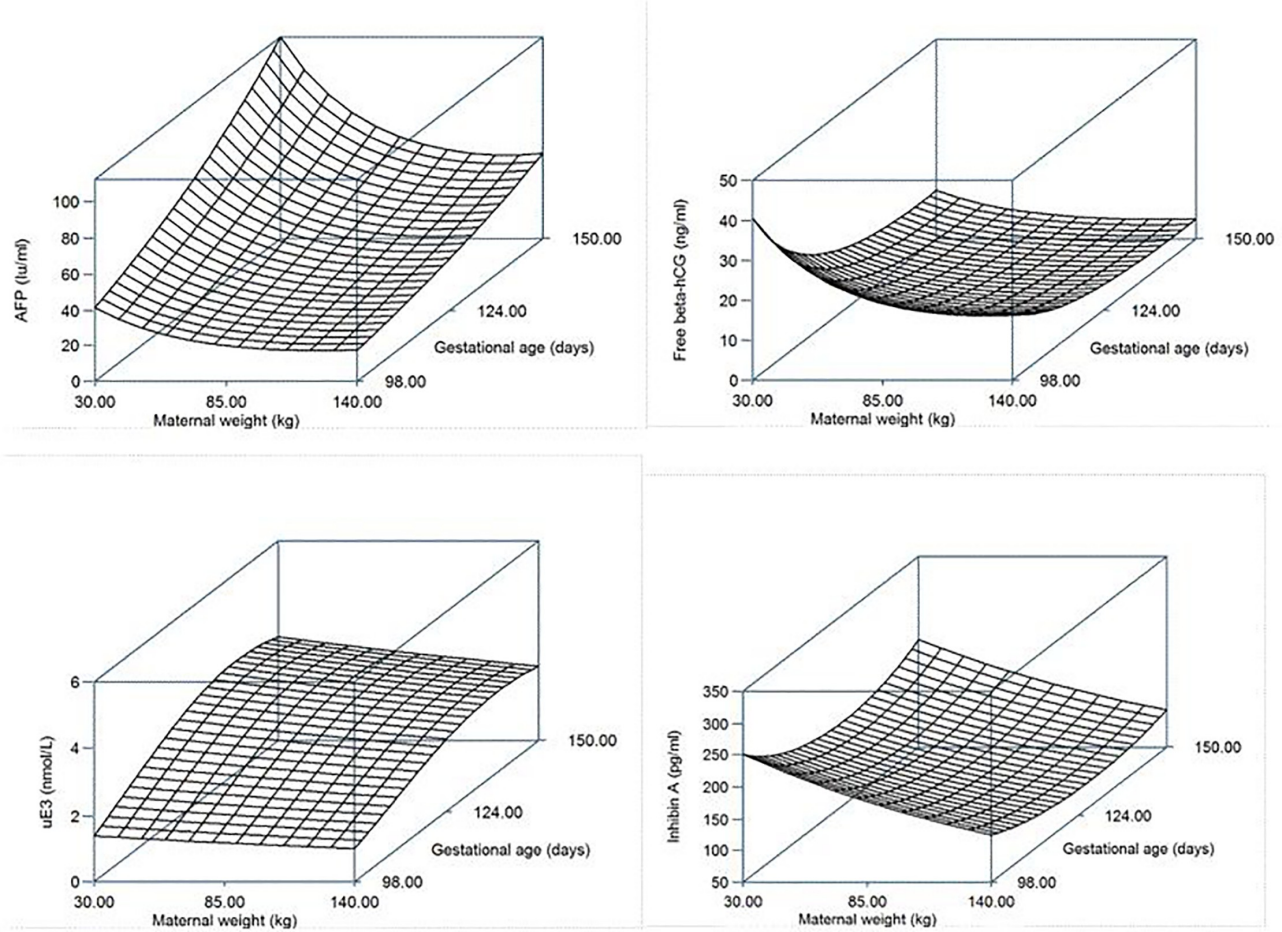
Surface models of predicted median MoMs of maternal serum quadruple levels constructed from the regression equations adjusted for gestational age (days) and maternal weight (kg) of Thai ethnicity pregnant women.

The surface plot models are demonstrated in Fig 3 and show the median maternal serum quadruple markers levels according to maternal weight and gestational age (days) of our population. Both serum AFP and uE3 levels increased with advanced gestation and were higher in patients with lighter maternal body weight. Serum free β-hCG levels decreased and then stabilized during the screening period with advancing gestational age and with smaller levels in heavy maternal weight patients. Serum inhibin A levels increased during the early screening period and decreased during the last period of screening, with a final slight increase during late gestation.

**Fig 4:**
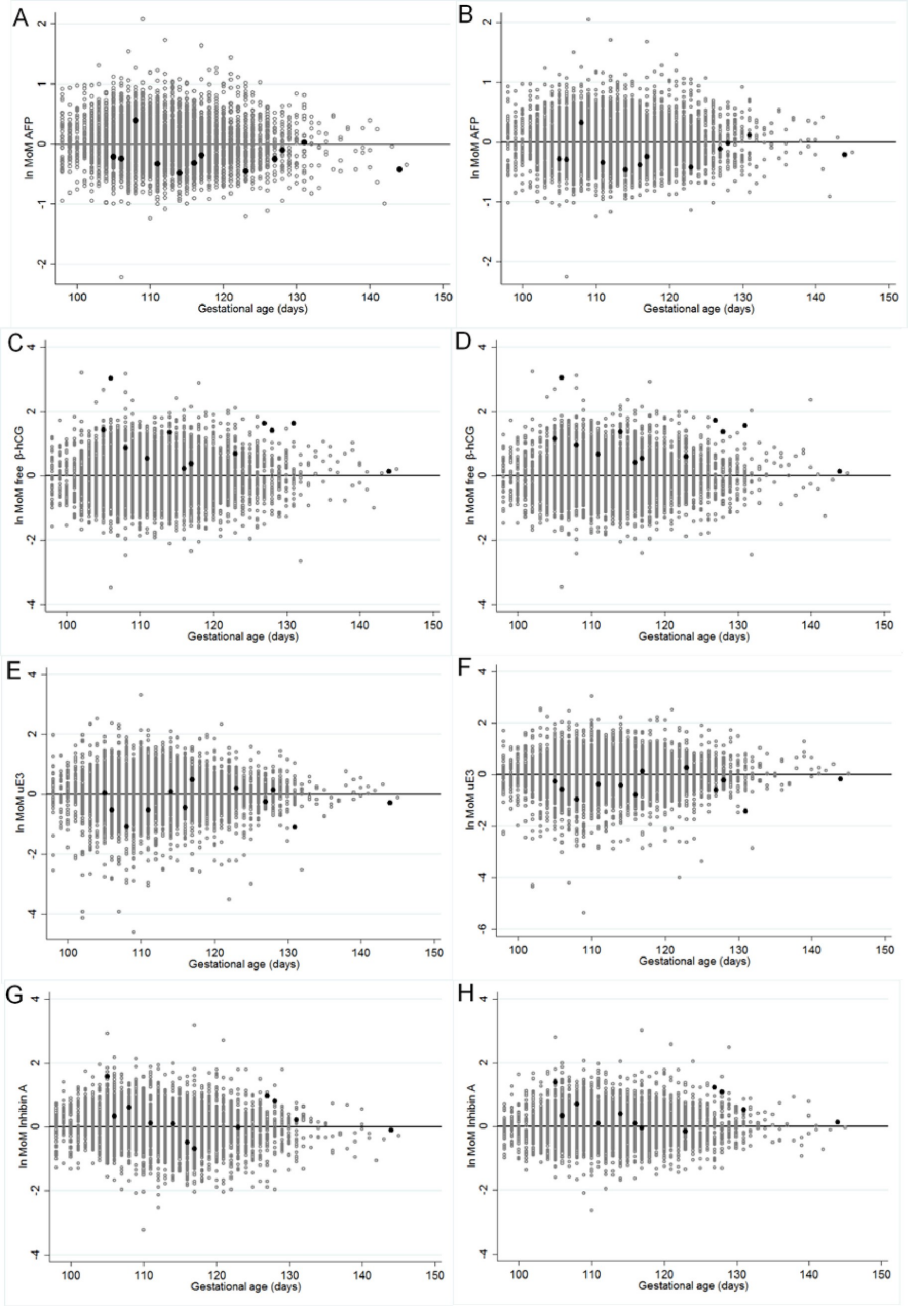
The scatter plots of log MoMs of maternal serum quadruple tests for screening for Down syndrome calculated with Western and Thai factors showing the differences in screening efficacy. Figures A, C, E and G show the scatter plots when using the Western European descent factor (WF) for screening. Figures B, D, F, H show the scatter plots when using the Thai factor (TF) for screening (grey spots show normal fetuses, black spots show Down syndrome fetuses).

The scatter plots of log MoM of each serum biomarker comparing normal fetuses and DS fetuses calculated using WF and TF are shown in Fig 4A–4H. The log MoMs of AFP and free β-hCG using either WF or TF had similar efficacies to detect DS fetuses, while for inhibin A and uE3, the predicted MoMs calculated with the TF had better accuracy in determining DS from normal fetuses than the WF reference range. For comparison, Table 2 shows the significant differences between the levels of median MoMs of maternal serum quadruple markers for normal and DS fetuses when automatically calculated with the WF and when calculated with our new TF regression equation. There were significant differences in the median MoM levels of AFP, free β-hCG and uE3 of normal fetuses calculated using the different ethnic factors. In the DS fetus group, there were significant differences in the median MoM levels of inhibin A and uE3 but no significant difference in the AFP and free β-hCG level.

**Table 2:** Comparison of median MoM levels of serum quadruple screening between the Down syndrome and normal fetus groups when calculated with Western European descent factor (WF) and Thai factor (TF).

Maternal Serum	Median MoM (IQR 1,3)					
	Down syndrome fetuses			Normal fetuses		
	WF	TF	*P* value	WF	TF	*P* value
AFP	0.78	0.77	0.479	1.02	1	<0.001
	(0.69,0.86)	(0.70,0.94)		(0.82,1.28)	(0.81,1.25)	
Free β-hCG	3.14	2.89	0.661	0.97	1	<0.001
	(1.57,4.64)	(1.76,4.35)		(0.64–1.47)	(0.67,1.52)	
uE3	0.76	0.57	0.028	1.06	1	<0.001
	(0.59, 1.11)	(0.50,0.82)		(0.73–2.17)	(0.67,1.50)	
Inhibin A	1.19	1.44	0.025	1.04	1	0.288
	(0.95, 2.05)	(1.11, 2.48)		(0.75, 1.41)	(0.75, 1.36)	

IQR: Interquartile range.

The performance of maternal serum quadruple screening calculated with WF in our population is shown in Table 3. When automatically calculated using the WF, 9 of 12 DS fetuses were identified, giving a detection rate of 75.0% with a false positive rate of 9.1%. However, compared with the predicted model calculated with the regression equation of TF, 10 of 12 DS fetuses were detected, giving a better detection rate of 83.3%, but with a slightly higher false positive rate of 9.6%. The false positive rates of the two calculation factors are comparable, but calculation with the TF gave a better detection rate.

**Table 3:** Comparison of quadruple maternal serum screening performance between using the Western European descent factor (WF) and the Thai factor (TF) for calculation.

	Screening performance %	
	Western European descent factor (WF)	Thai factor (TF)
Detection rate	75.0	83.3
Specificity	90.9	90.4
False-positive rate	9.1	9.6
Positive predictive value	1.8	1.9
Negative predictive value	99.7	100

## Discussion

This study was conducted using a prospective design to evaluate quadruple serum screening in 2 referral centers in the south of Thailand. All the participants were Thai nationals who attended the antenatal care clinic in one of these two tertiary care hospitals. All 5,515 participants delivered in one of these hospitals, and we had no cases lost to follow up. The average maternal age at screening was 29.1 years (14.0, 46.0), with 11% of the patients ≥35 years and thus defined as high-risk patients. The participant age groups were the same as those used in other developing countries [16]. The highest number of screenings were performed in the 15^th^ and 16^th^ weeks of gestation which also was their first time attending antenatal care.

The median MoMs of maternal serum free β-hCG and inhibin A of the DS fetuses in this study were higher than the normal fetuses, while the serum levels of AFP and uE3 were lower in the DS fetuses than in the normal fetuses (Table 2). These results are in concordance with the levels of maternal serum quadruple markers of DS fetuses found in studies from Western countries [11, 12] and one study done in an Asian population [10]. However, by calculating the median serum quadruple marker levels with the regression equation adjusted for the Thai reference ranges of our population, we demonstrated significant differences between the TF and WF calculations in normal and DS fetuses, as shown in Table 2. Theoretically, the adjusted MoM of normal fetuses should be close to 1.0 MoM, but when the adjusted normative median was constructed based on the Thai reference values the median MoMs of our Thai specific model were closer to 1.0 than the MoMs adjusted for the Western model (Table 2).

Other studies have reported similar results, that the median MoMs of the maternal serum quadruple test of normal Asian fetuses were higher than those of Western populations [17–19]. Thus, we recommend not using the WF model as a standard cutoff in quadruple screening for our and other Asian populations. Calculating a new regression equation adjusted for the maternal age and gestational age of our ethnic factor gave the optimal model estimation of serum levels for pregnant Thai women, as shown in Fig 3. In gestational age beyond the first trimester, AFP, inhibin A and uE3 were generally higher, but the concentrations decreased with higher maternal weight, while free β-hCG had lower concentrations and lower maternal serum with increasing maternal weight. This evidence supports our findings that maternal weight adjusted for median MoM affects the maternal serum levels. Thus, maternal weight adjustment for serum calculation has an important role as this will vary among nations [7, 20]. Moreover, our study and previous studies have shown that race and ethnic factors have an important effect on serum concentrations both in DS and normal fetuses throughout the gestational period [7, 10–12, 20]. Another study from our institution reported the same evidence from first trimester serum screening for DS [21]. Maternal serum quadruple markers do not have universally applicable levels for screening for DS in all races and ethnicities, and have also been reported in other studies which found differences in normative median serum levels in subgroups of the same country or ethnicity [14, 20].

Our study determined predicted median MoMs corrected for maternal body weight, gestational age in days and the ethic factor of our study population. When comparing the levels of serum biomarkers calculated with TF compared with the built-in WF in the standard machine analysis of normal fetuses, there were significant differences in AFP, free β-hCG and uE3 maternal serum levels between the two models, as shown in Table 2. These results were similar to another study in Thailand using the maternal serum triple test when maternal weight and gestational age (in weeks) were corrected in a similar fashion [20]. However, the different levels of serum markers were greater in the DS group when the two models of calculations were compared.

When the MoMs of each serum level in the DS fetuses are compared as shown in Table 4, our study’s AFP levels were not different between the two ethnic factor calculations. Compared with other studies, the MoM of AFP was not significantly different from other Asian and Western population studies (Table 4). However, there was a notable difference in the level of AFP between our study and that of Kaewsuksai [14], which involved the same southern Thai population, but the serum levels in that study were calculated with the built-in WF of the machine. Also, the MoM of AFP of Kaewsuksai’s study was much higher than those reported from studies in Western populations [11, 12]. The MoM level of free β-hCG of DS fetuses in our study was higher than those of other Asian and Western studies (Table 4). In the same way, a significantly high free β-hCG level was found in Kaewsuksai’s study when calculated with WF. From these results, we can conclude that the free β-hCG level of pregnant Thai women is higher than in other ethnicities that have been reported even after adjusting for the ethnic factor. The MoM uE3 level in our study was slightly lower than in other studies of Asian and Western ethnicities. The MoM level of inhibin A in our study was significantly lower than in previous studies of Asian and Western ethnicities. According to the different MoMs of DS fetuses found in comparisons of previous studies in Asian and Western populations, we recommend that DS cutoff values should be constructed based on individual populations. Furthermore, the predicted median among normal fetuses and DS fetuses also showed a significant difference between the two calculation methods. Calculations with the Western ethnicity model overestimated the free β-hCG and uE3 levels, and underestimated the inhibin A level, thus using the automatic calculations based on the Western ethnicity model built in to most analyzers is not sufficiently accurate for our Thai population.

**Table 4:** Comparison of median MoMs in Down syndrome fetuses between different races and ethnicities.

Study	Number of Down syndrome fetuses/screening participants	Maternal serum quadruple (MoM)			
		AFP	free β-hCG	uE3	Inhibin A
Our study	12 /5,155				
• Thai factor		0.77	2.89	0.57	1.44
• Western European descent factor		0.78	3.14	0.78	1.19
Kaewsuksai	4/2,375	1.2	3.95	0.73	2.96
Tana	35/20,229	0.63	1.72	0.65	-
Shaw	11/21,481	0.87	2.34	0.77	2.16
SURRUS	88/46,193	0.74	2.05	0.77	2.54
FASTER	87/35,236	0.74	1.91	0.61	2

For DS screening in the second trimester, either the quadruple or quad test has been an acceptable option in both developed and developing countries [5, 6, 8]. The screening period for these tests is between 14 and 22 weeks gestation, with reported detection rates of between 78 and 85% [10–14]. The reported risk cutoff varies among studies between 1 in 250 to 300 [10–12, 14, 22–24], and also there are differences in false positive rates of between 4.4 and 8.6% [9–14]. In this study, we chose a risk cutoff of 1:250 or greater because this is widely used [10–14]. Using the WF in our patients gave a detection rate of 75% with a false positive rate of 9.1% and overall accuracy of 90.1%, which was a lower detection rate than in previous studies but comparable with the study of Kaewsuksai et al. [14]. In our study, although we had a similar study population and we used a similar analytical technique process as the Kaewsuksai study, when the quadruple serums were re-calculated using the TF in our regression equation, the diagnostic performance was better than using the MoMs calculated with the WF and comparable with previous studies [10–13]. The detection rate was higher with only a minimal increase in the false positive rate using the TF than the WF calculation in our study, as shown in Table 3. This was in contrast to a study by Tana et al. [20], which was also done in Thai pregnant women but reported the detection rate of triple serum markers, and which found that using the ethnic-specific factor resulted in a remarkable decrease in the false positive rate although the detection rate was still the same. Many studies in Asian populations have found that using ethnic factors had an impact on the results of quadruple serum screening [7, 10, 25–28]. Our study and evidence from previous studies support the idea that using an ethnic-specific reference range has a significant impact on the diagnostic performance in each population [29–31]. However, our study showed different ranges of maternal serum within the same ethnic group unlike other studies [14, 20]. Further investigations are needed to confirm whether various lab settings need their reference ranges adjusted for the screening test.

Our study was a prospective design, with all cases accounted for at the end of the study period. All participants’ gestational ages were corrected with ultrasound before 20 weeks, not only estimated from clinical examinations or last menstrual period, which can give a wrong gestational age at screening. All of the study participants were of Thai ethnicity; there were no patients with mixed race or other ethnicity, unlike other studies [11, 12]. All the newborn babies were evaluated by a neonatologist in one of the hospitals, and baby karyotyping was performed in all cases of abnormal chromosomes or when a genetic syndrome was suspected. All blood specimens were directly sent to the laboratory of primary institute (Songklanagarind Hospital) which had monthly quality control checks, thus potential problems or errors due to specimen transfers were eliminated, unlike other studies [32]. The notable strengths of our study were (1) the model created from our regression equation was adjusted for maternal weight and gestational age in days, and (2) the study was done in a homogeneous population.

This study’s main limitation was the moderate number of participants; the sample size of our study was quite small during the late screening period. The rates of maternal serum levels at the late screening periods may not sufficiently represent the normal distribution pattern at these screening periods. However, we had a sufficient number of DS fetuses to compare our results with other studies. The study required a long period because we included only patients who attended antenatal care in the tertiary centers to allow us to control all important influencing factors. In our study, we did not evaluate the screening performance of other aneuploidies. Also, we had only a small number of advanced maternal age participants. However, the average maternal age of our study was similar to the average maternal age in other developing countries which also use quadruple maternal serum screening in the second trimester, due to the late attendance at antenatal care of most pregnant women in developing countries, which also have a lack of facilities and funding and are also generally unable to afford the more expensive cell-free fetal DNA test.

Our study has important implications concerning the ethnic factor in calculating the efficacy of the quadruple test but is especially important for our country or similar countries where we provide the test for free, thus limiting the number of false positives with acceptable detection rate are important in order to avoid placing unnecessary costs on the healthcare budget. This study could also be used as a second trimester screening model for use in other hospitals in Thailand and other developing countries.

## Conclusions

The serum quadruple test had a moderate detection rate as a universal screening test for Down syndrome in the second trimester in our study. Based on our study, we recommend that regression equations adjusted for maternal weight and gestation in days for ethnic-specific factors are needed to establish appropriate reference ranges for normal and Down syndrome fetuses. Individual ethnic-specific factors for maternal serum levels would facilitate optimizing the efficacy of Down syndrome screening and reduce unnecessary burdens on the health care budgets of low resource countries.

## Supporting information

S1 Dataset Dataset of quadruple test used for our analyses. This dataset excludes miscarriages and other chromosomal abnormalities other than Down syndrome.
(XLSX)
